# The impact of the AMV on Eurasian summer hydrological cycle

**DOI:** 10.1038/s41598-020-71464-2

**Published:** 2020-09-02

**Authors:** Dario Nicolì, Alessio Bellucci, Doroteaciro Iovino, Paolo Ruggieri, Silvio Gualdi

**Affiliations:** 1 Fondazione Centro Euro-Mediterraneo Sui Cambiamenti Climatici (CMCC), Bologna, Italy; 2 Ca’Foscari University of Venice, Venice, Italy; 3 Department of Physics and Astronomy, University of Bologna, Bologna, Italy; 4 Istituto Nazionale Di Geofisica E Vulcanologia (INGV), Bologna, Italy

**Keywords:** Climate sciences, Atmospheric science, Hydrology

## Abstract

Impact studies of the Atlantic Multidecadal Variability (AMV) on the climate system are severely limited by the lack of sufficiently long observational records. Relying on a model-based approach is therefore mandatory to overcome this limitation. Here, a novel experimental setup, designed in the framework of the CMIP6-endorsed Decadal Climate Prediction Project, is applied to the CMCC climate model to analyse the remote climate impact of the AMV on the Northern Eurasian continent. Model results show that, during Boreal summer, an enhanced warming associated to a positive phase of the AMV, induces a hemispheric-scale wave-train response in the atmospheric circulation, affecting vast portions of Northern Eurasia. The overall AMV-induced response consists in an upper-tropospheric anomalous flows leading to a rainfall increase over Scandinavia and Siberia and to an intensified river runoff by the major Siberian rivers. A strengthening of Eurasian shelves’ stratification, broadly consistent with the anomalous river discharge, is found in the proximity of the river mouths during positive-AMV years. Considering that Siberian rivers (Ob’, Yenisei and Lena) account for almost half of the Arctic freshwater input provided by terrestrial sources, the implications of these findings for decadal variability and predictability of the Arctic environment are also discussed.

## Introduction

The variability of Northern Eurasian climate has direct implications for the hydrological cycle of the Arctic^1,2^. Almost 10% of the total freshwater input into the Arctic Ocean derives from terrestrial sources, influencing sea-ice formation and sea surface salinity at different timescales^3,4^.

High-latitude precipitation is one of the drivers of the Arctic rivers discharge, even if the direct link between rainfall and river streamflow is still unclear^5,6^, due to the concurrence of several additional factors which may have implications for the freshwater inflow: snowmelt, permafrost degradation, reservoir and dam regulation, fire frequency and human activities^7^. However, understanding the origins of the regional scale, decadal variability of precipitation remains a challenging issue, which may disclose some degree of predictability, potentially improving the quality of climate predictions. Several studies have focused on the winter period, invoking a role for the Siberian High, Northern Hemisphere Annular Mode and sea surface temperature (SST) variability in the North Atlantic and Pacific Ocean^8–12^. Berg et al.^13^ claim that, at daily timescale, increasing surface temperature, the Clausius–Clapeyron law prevails in the modulation of the large-scale precipitation, whereas moisture transport and availability are the keys to explain summer rainfall. Different physical processes require different approaches for winter and summer analysis, given also the strong climate seasonality of the high-latitude regions^5,14^.

On the interannual timescale, summer precipitation is still driven by the convergence of large-scale moisture fluxes over eastern Siberia since the evapotranspiration variability is negligible over that timescale^15^. The early work of Peng and Mysak^16^ proposed that negative anomalies of western Siberian summer precipitation and river runoff can be interpreted as the lagged response to the extratropical SST warming of the North Atlantic Ocean during wintertime. Another study focuses on the eastern and western portion of Siberia, showing opposite spatial patterns in precipitation field due to an oscillating zonal dipole in atmospheric circulation with a 6–8-year timescale, in agreement with the dry/wet regimes of the region^15^. This seesaw pattern may be induced by a quasi-stationary Rossby wave, which crosses the Eurasian continent and favours anomalous meridional-winds formation^17^.

Given the lack of adequately-long observational records of river discharge and precipitation over Northern Eurasia, very few studies focus on the decadal-scale variability. Among the others, Zhang et al.^18^ reveal a positive trend for the poleward moisture transport (2.6% per decade) and river runoff (1.8% per decade) under a warming climate scenario. They found the high-latitude wetting trend which may depend from an anomalous Icelandic Low, advecting moisture from the North Atlantic and transporting it along the northern sector of Eurasia^19^, as also detected by Fujinami et al.^5^ at interannual time scale.

Sun et al.^20^ (hereafter S15) show a strong correlation between Siberian warm-season precipitation and the Atlantic Multidecadal Variability (AMV), a low frequency variability signal affecting the basin-wide North Atlantic SSTs. Based on observations and numerical simulations, performed with the atmospheric general circulation model SPEEDY^21^, the authors found that the AMV positive phase induces an eastward wave train over Eurasia. The altered circulation promotes a northward transport of moisture around 100°E from the North Pacific Ocean, featured by an anomalous anticyclone over North-eastern Asia, which leads to an increase of precipitation over Siberia.

Here, the imprint of the AMV on the Northern Eurasian multidecadal variability is inspected via a novel experimental framework, specifically designed to isolate the influence of individual climate variability modes, using idealized model settings. We adopt the experimental protocol for the CMIP6-endorsed Decadal Climate Prediction Project (DCPP, component C), detailed in Boer et al.^22^, with the aim of analysing long-timescale climate variability and attributing their regional impacts.

Compared to S15^20^, we have used a state-of-the-art coupled general circulation model, which accounts for active interactions between the climate components, allowing a wide understanding of the observed low-frequency variability of precipitation. The adopted experimental setup consists in locally nudging a positive (negative) AMV-like SST anomaly pattern over the North Atlantic Ocean to simulate the warm (cold) phase of the AMV, while leaving the remaining parts of the system free to evolve (for further details see the “Method” section).

This kind of approach allows a cleaner attribution of the observed low frequency climate variability signals to the AMV, since all the climate components feedback to the imposed signal. Similar, pre-CMIP6 implementations of this methodology have been used in previous analyses^23,24^, targeting specific processes at the global and regional scale. Here, this novel methodology is applied to assess the global climate response to the AMV anomalies. Moreover, we address the role of AMV on the multidecadal variability of the Northern Eurasian hydrological cycle, with particular emphasis on the Siberian region, and the links with the Arctic domain.

## Results

### The global response

In this section, the global climate impacts of the AMV anomalies are presented. These are diagnosed in terms of difference patterns between the AMV+ and AMV− ensemble means, calculated for different key variables, assuming a linear response of the climate system to the imposed North Atlantic SST perturbation^23^. In the calculation of the mean values, years 2 to 10 from each hindcast simulation are considered—i.e. neglecting the first year of the simulation—in order to reduce the residual adjustment after initialization. The statistical robustness of the results is evaluated by means of a two-tailed Student's t test, with a 95% confidence level of significance. Here, we focus on the boreal summer season (JJAS) for consistency with the regional analysis illustrated in the following section. The response in winter season is presented in the supplementary material (Fig. S.1).

**Figure 1:**
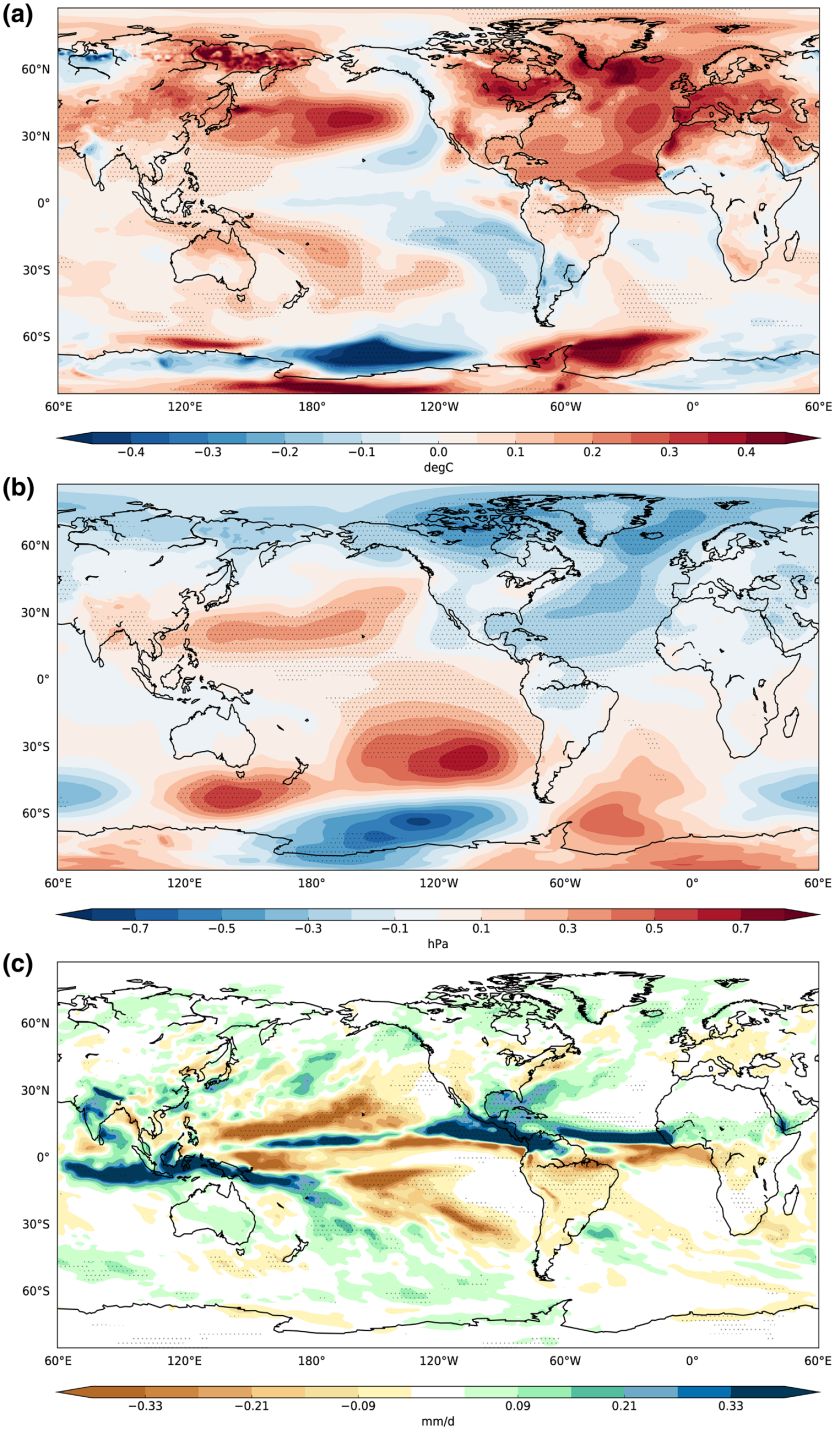
(**a**) Boreal summer (JJAS) differences between the last 9-year ensemble mean of the positive and negative phases of the AMV experiments for 2-m temperature (°C), (**b**) Sea Level Pressure (hPa) and (**c**) Precipitation (mm/d). Dotted regions display significant values (student's t test with 95% confidence level). This figure was plotted using Python 2.7.12 (https://www.python.org/download/releases/2.7/).

Figure 1 shows the linear response obtained for two-meter temperature field. Over the North Atlantic Ocean, model anomalies are consistent with the imposed AMV pattern (Fig. S.2), with a significant warming over the entire basin and local maxima (up to 0.5 °C) over the subpolar gyre, Eastern Atlantic and the tropical belt (Fig. 1a). The amplitude of the anomalies is not twice the amplitude of the observed patterns, as one may expect from computing the difference between the AMV+ and AMV− responses, because the high frequency of the ocean–atmosphere coupling tends to counteract the restoring efficiency. On the other hand, a decrease of the coupling frequency or a too strong restoring to the prescribed SST may lead to spurious drifts. A significant warming signal is also found over South-eastern Europe, the Mediterranean basin and a wide region encompassing the Arabian Peninsula and Eastern Africa, consistent with previous findings^25–28^. Over the Middle East, temperature increase is attributable to the observed AMV-induced surface pressure gradient which locally transports warm moist air over the dry, hot region^29^.

The North American continent features a surface-temperature zonal dipole, with warm (cold) anomalies over the Ontario (Alaskan) region. A positive AMV pattern leads to warmer temperatures over South-Western U.S., Central America and North-Eastern Brazil, broadly close to the observations^25,27^.

The observed AMV-related impacts are well reproduced by the model, not only in the surrounding areas of the Atlantic. A slight but significant warming (0.1–0.2 °C) affects the Western Tropical Pacific (WTP), the Maritime continent and the Northern part of Indian Ocean, in agreement with the existing literature^30–32^.

The Pacific Ocean response to the imposed AMV perturbation closely resembles the negative phase of the Interdecadal Pacific Oscillation (IPO), with warm lobes over the extra-tropics, rimmed by cold SST anomalies over the tropics and bordering the western coasts of the American continent. Pacific sea level pressure (SLP) increases in response to the reduced North Atlantic anomalies (Fig. 1b), suggesting that a warm phase of the AMV could alter the Walker circulation^23,33,34^. Along the equatorial Pacific, easterly surface winds are reinforced (weakened) through Western (Eastern) part of the basin, allowing (preventing) heat transfer from the ocean into the atmosphere (Fig. S.5). SST changes are consistent with the ocean subsurface temperature anomalies for an equatorial cross-section (Fig. S.6), revealing a cooling in the central and eastern side of the basin and a warming in the Western Pacific. The detected subsurface temperature zonal dipole suggests an upwelling of cold waters induced by the intensification of the easterlies.

Changes in atmospheric circulation are characterized by negative SLP anomalies, which involve the most part of the Northern Hemisphere with minima centred in the Nordic Seas, Baffin Bay and North Atlantic Ocean (Fig. 1b). Interestingly, the European sector shows a reduction of SLP, associated with warm and dry anomalies, which is partially in line with previous studies^25,26^.

Marked changes in precipitation field are evident along the Tropics, with positive anomalies straddling most of the tropical belt (Fig. 1c). These anomalies are consistent with a northward shift of the Inter-Tropical Convergence Zone (ITCZ) likely driven by the inter-hemispheric energy imbalance associated with the North Atlantic warming^23,35^. On the western side of the Pacific Ocean, the Maritime continent experiences enhanced rainfall with anomalies suggesting a South-Westward shift of the South Pacific Convergence Zone, a band of low-level convergence, cloudiness and precipitation. Wetter conditions over Indian peninsula may indicate a role for the AMV in modulating the Indian summer monsoon rainfall as suggested by previous works^24,36,37^. A significant reduction of rainfall is also found over off-equatorial regions, including Central and Eastern tropical Pacific, California and Amazonia region, while above-normal precipitation marginally involves the northernmost regions, above 60°N, including the Baffin Bay, the Nordic Seas and the northern part of the Eurasian continent. In particular, a significant increase of precipitation covers the Siberian region, potentially leading to enhanced freshwater inflow from the local rivers into the Arctic Ocean. A closer inspection of the mechanism behind this response is given in the next section.

### Case study: Siberian precipitation

**Figure 2:**
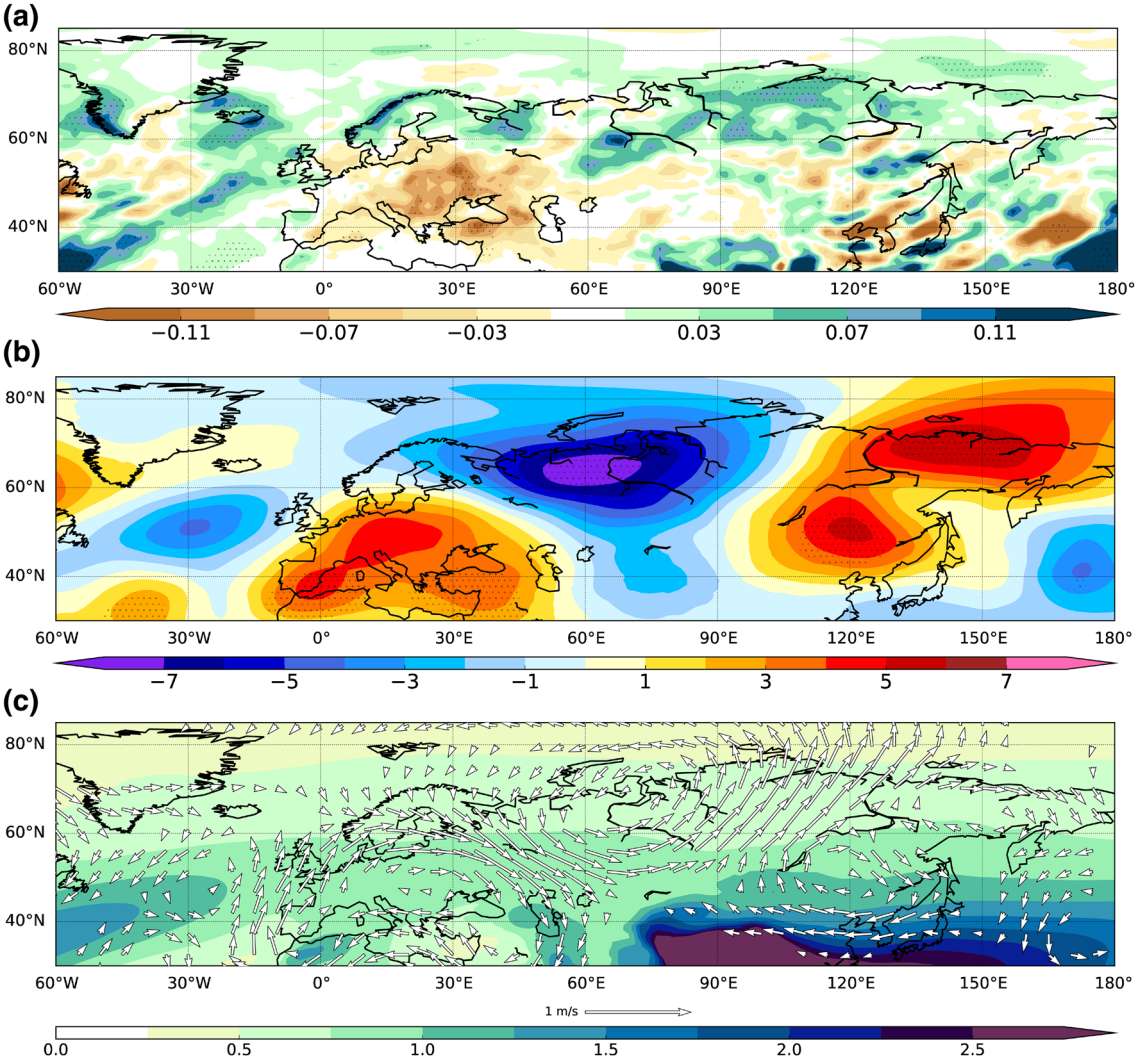
May-to-August differences between the last 9-year ensemble mean of the positive and negative phases of the AMV experiments for (**a**) precipitation (mm/d), (**b**) departure from the zonal mean of the geopotential height at 300 hPa (m) and (**c**) climatological specific humidity (g/kg) and wind anomaly (m/s) at 500 hPa (shading and vectors). Dotted regions display significant values (Student's t test with 95% confidence level). This figure was plotted using Python 2.7.12 (https://www.python.org/download/releases/2.7/).

We investigate the influence of the AMV on the Northern Eurasia precipitation and its impact on the freshwater balance of the Arctic Ocean. Siberian rainfall peaks in July^15,38^, and July precipitation accounts for more than 40% of total annual precipitation in the area^5^ broadly in phase with the climatological maximum of the Siberian rivers discharge^39^. The early-summer (from May to August) total precipitation anomaly, in terms of the residual patterns between the ensemble means of the positive and negative phase of the AMV, is shown in Fig. 2a. A significant increase of rainfall over the northern part of the Eurasia is found: an anomalous meridional dipole characterizes the European continent, with wetter condition over Scandinavia (+ 0.1 mm/day) and drier condition over the eastern Europe (− 0.1 mm/day), resembling the impact of the warm AMV phase found in previous observational^26^ and modelling studies^23^.

Interestingly, the largest signal is found over Siberia, where rainfall increases up to 10% of its model climatology during positive-AMV years, consistent with the aforementioned S15^20^ work and observations (mean precipitation of the region is 1.59 mm/day, according to GPCC dataset^40^). A number of 10 out of 32 members are sufficient to obtain statistically significant rainfall (not shown), following Deser et al.^41^.

Net precipitation (P–E) is often used in this kind of analysis to take into account the contribution of the evaporation. However, evaporation rate is generally low at high latitudes, except for lakes and wetlands^42^. This is further corroborated by comparing net precipitation (Fig. S.3) with the total precipitation (Fig. 2a) of the AMV composite patterns.

The AMV-induced changes of the Siberian early-summer precipitation may be justified by atmospheric circulation anomalies. We show the zonally asymmetric geopotential-height anomaly at 300 hPa (Z300*) to emphasize gradients linked to changes in atmospheric flows. The AMV composites for Z300* (Fig. 2b) reveal that the multidecadal warming of the North Atlantic SST excites an east–west northern-hemispheric wave train. A positive anomaly covers the Euro-Mediterranean region, which may partially explain the aforementioned significant decrease of total precipitation in the south-eastern Europe (Fig. 2a).

The Siberian area features a zonal dipole with positive and negative anomalies centred over the eastern and western part of the region, respectively, indicating that the AMV induces a southerly flow in between. This becomes clearer looking at the 500-hPa anomalous wind field (Fig. 2c, vectors) in which a northward flow is recognizable around 100°E, in agreement with the S15^20^ analyses. This flow lies at the convergence between a continental westerly flow and an easterly circulation anomaly flowing from the Pacific sector. The climatological specific humidity pattern at 500 hPa (Fig. 2c, shading) features a pronounced meridional gradient in south-eastern continental Asia and sub-tropical Pacific.

**Figure 3:**
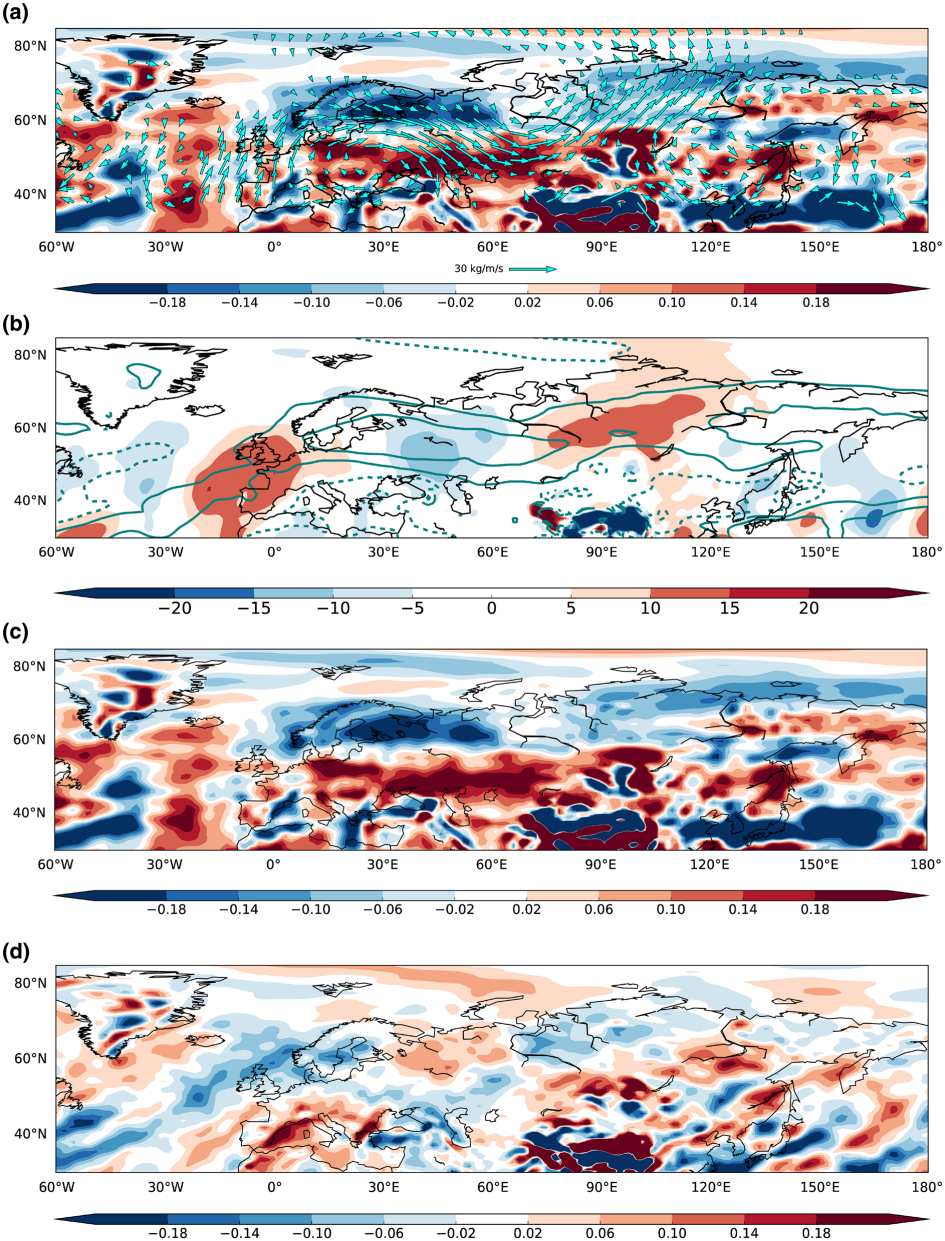
May-to-August differences between the last 9-year ensemble mean of the positive and negative phases of the AMV experiments for (**a**) vertically-integrated moisture flux (vectors, kg/m/s, vectors < 4 kg/m/s not shown) and its divergence (shading, mm/day), (**b**) zonal (contours, interval between − 15 and 15 kg/m/s) and meridional (shading) vertically-integrated moisture flux (kg/m/s), (**c**) divergence of zonal vertically-integrated moisture flux (mm/day), (**d**) same as (**c**) but for meridional component. This figure was plotted using Python 2.7.12 (https://www.python.org/download/releases/2.7/).

These findings motivate a more detailed moisture budget analysis. The AMV-composite for linear contribution of the vertically-integrated moisture flux is shown in Fig. 3a (vectors) as the combination of the monthly background moisture gradient and the AMV-induced anomalous wind to highlight the net meridional advection of moisture from low to high latitudes. The resulting pattern resembles the composite flow field (shown in Fig. 2c), with an eastward wave-like flux from the North Atlantic Ocean towards the western Russia and a second anticyclonic flow centred over Mongolia, both converging over Siberia, around 100°E.

To clarify the mechanism at stake, we focus on the divergence of the vertically-integrated moisture flux, hereafter MFD (Fig. 3a, shading areas). Moisture flux convergence (associated with negative MFD anomalies) is found over a zonally elongated belt straddling the Eurasian continent north of 60°N, with local MFD minima over Scandinavia and Siberia, where maximum precipitation anomalies are also found (Fig. 2a), suggesting a tight relationship between the detected rainfall enhancement and the increase in moisture convergence over those areas. The anticyclonic cell, placed over 100–140°E and 40–60°N, is mainly characterized by positive MFD which supports our previous conjecture about its contribution in transporting moisture from the Pacific Ocean towards the high latitudes.

The individual zonal and meridional components of the vertically-integrated moisture flux are examined^43^. Strong positive anomalies of zonal moisture transport (Fig. 3b, contours) prevails over the Scandinavian region compared to meridional contribution (Fig. 3b, shading), counteracting the weak northerlies, which are linked to dry conditions, and supporting a strengthening of the mean westerlies (Fig. 2c, vectors). This is further corroborated by the MFD of the single contributions, where zonal convergence (negative anomalies in Fig. 3c) is broadly 2 times the meridional one (Fig. 3d) over Eastern Scandinavia and Western Russia.

Meridional moisture flux field (Fig. 3b, shading) shows an anomalous seesaw pattern along the Arctic side of the Eurasian continent. The wider anomaly is located over the Siberian region from 70°E to 130°E and latitudinally extended from 40°N to 70°N, implying the poleward recall of moisture from lower latitude.

Heavier and more significant precipitation anomalies emerge over the Siberian region (Fig. 2a), compared to Northern Europe. The contributions of the single components suggest that meridional advection is not negligible over this area, since same-magnitude convergence occurs (Fig. 3c,d), reinforcing the idea that the AMV-induced rainfall can be traced back to the advection of moisture from the Atlantic and Pacific Ocean basins.

The Ob’, Yenisei and Lena rivers accounts for about 46% of the input of freshwater into the Arctic provided by terrestrial sources and their watersheds (795 × 10^4^ km^2^) cover more than 60% of the catchment areas of all Eurasian Arctic rivers^18^. Therefore, the increased rainfall over Eastern Siberia may significantly contribute to a freshening of the Arctic Ocean via an enhanced river discharge. For instance, Yenisei and Lena runoffs contribute to about half of the total Arctic-river discharge in June^39^.

**Figure 4:**
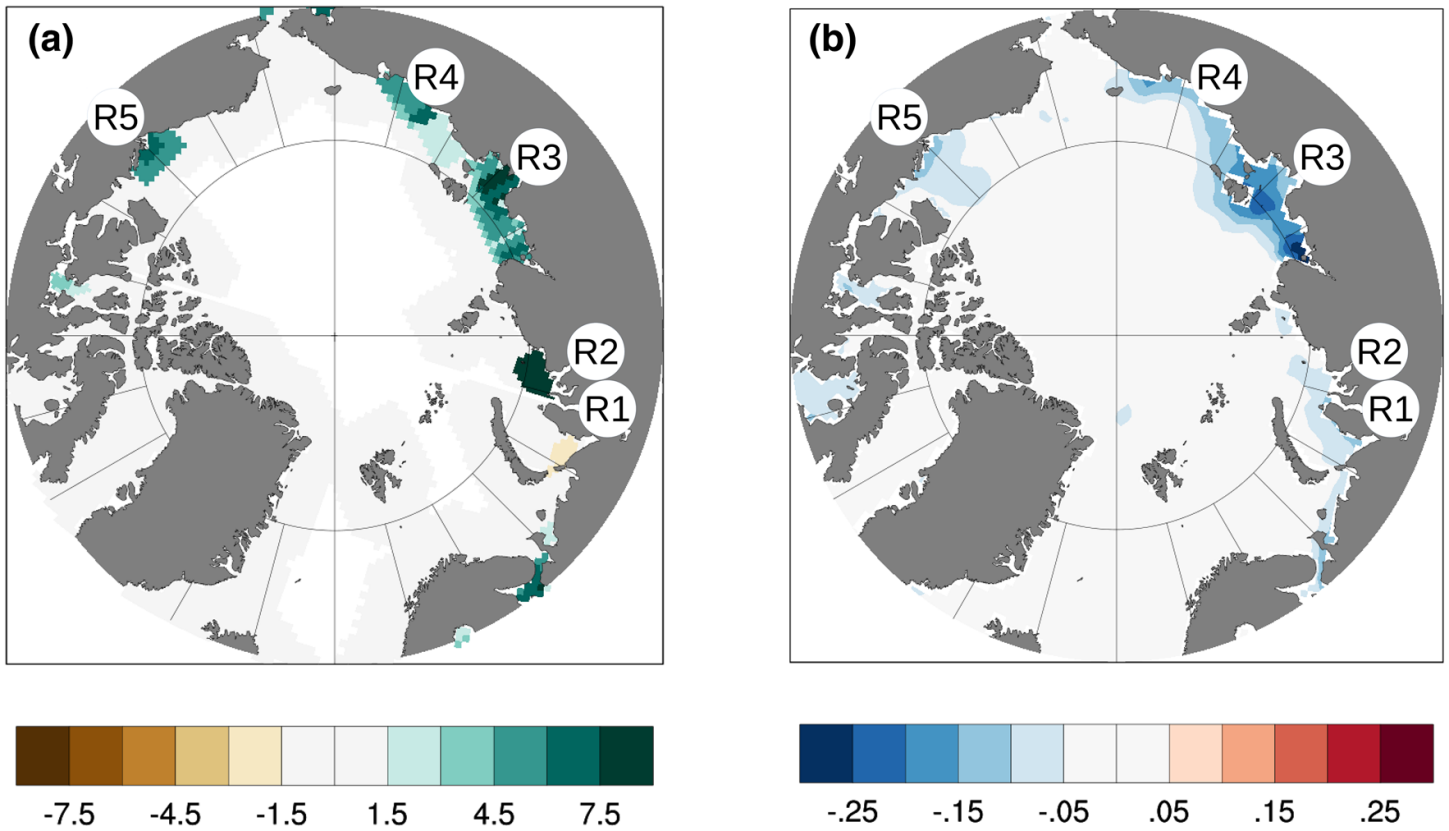
May-to-August differences between the last 9-year ensemble mean of the positive and negative phases of the AMV experiments for (**a**) river runoff (10^−6^ kg/m^2^/s) and (**b**) ocean potential density (kg/m^3^). Displayed rivers are: Ob’ (R1), Yenisei (R2), Lena (R3), Kolyma (R4) and Mackenzie (R5). This figure was plotted using Python 2.7.12 (https://www.python.org/download/releases/2.7/).

Figure 4a shows the AMV-composite of river runoff, here expressed as the freshwater flux at the river mouth on the top oceanic level. From May to August, Ob’ and Yenisei as a whole release 2.74 km^3^ month^−1^ of anomalous AMV-associated freshwater into the Arctic Ocean (i.e. 3.33% of its model climatological value). Furthermore, a positive phase of the AMV is associated with a surplus of discharge in Lena river (featuring the largest annual discharge among the Arctic rivers^39,44^), of 4.23 km^3^ month^−1^ (i.e. 3.85% more than its model average over the same period). Other noticeable sources of freshwater are provided by the Kolyma river, in the Eastern Siberia, and the Mackenzie river, in northern Canada.

A surplus of the Eurasian rivers freshwater inflow affects the ocean stratification of the Siberian marginal seas. Figure 4b shows the anomalous potential density averaged over the upper 50-m layer. Negative anomalies are evident along the Arctic coast, with local minima close to the river mouths. In particular, a 0.3 kg m^−3^ decrease is found in the Laptev and East Siberian seas, in correspondence of the Lena and Kolyma rivers mouths. Lower amplitude anomalies cover also Kara Sea, close to Ob’ and Yenisei rivers’ mouths, and surrounding areas of the Mackenzie river end. The apparent reduced impact of these rivers on the surface salinity budget, compared to what found for the other Arctic rivers, is likely due to fast vertical mixing processes which dominate the shallow Kara Sea during the warm season^45^. A more intense river freshwater and a stronger stratification, linked to local surface freshening (Fig. S.7), leads to an increase of heat content in the sub-surface layer (Fig. S.8) and a reduction of sea-ice cover during positive AMV years (Fig. S.9) over the shelf^46–48^.

## Discussion

The idealized DCPP-C simulations analysed in this study stress the role of the AMV in driving significant changes in surface temperature, precipitation and synoptic atmospheric circulation at both global and regional scale.

As expected, the largest response, marked by a strong seasonal dependency, is found over the Northern Hemisphere. The AMV-induced changes in boreal summer (from June to September) precipitation are consistent with a northward shift and a strengthening of the ITCZ, leading to increased rainfall over the Atlantic equatorial belt, including the semiarid Sahel region and Central America, in agreement with previous findings^25,27^. This result corroborates the idea described by Wang^49^ of an ITCZ-shift positive feedback, in which enhanced rainfall induced by a warm AMV-phase decreases dust concentration over the Tropics, thus further increasing North Atlantic SST. North-East Brazil directly experiences the ITCZ shift with dry summer anomalies during a positive AMV as seen in earlier works^50,51^. Moreover, Western Europe exhibits an anomalous warming at the surface in agreement with the positive trend observed during the last decades^26^, fostering the link between AMV and the extreme dry periods in that region.

The AMV perturbation also affects remote areas of the globe such as the Indian region where above-normal convective precipitation emerges during the summer monsoon rainfall season. Over the Pacific Ocean the AMV anomalies project the SSTs towards a negative IPO-like pattern, featured by warmer than average mid-latitudes and cooler than average equatorial band, associated with a La Nina-like pattern in the tropics. Notably, the detected AMV-IPO connection supports the idea that the "hiatus" in the global mean temperature rise, occurred in the early 2000s, is a lagged Pacific response to a cold-to-warm AMV phase transition^33,52^.

In a second part of this work, we assess the potential AMV impact on early-summer (May-to-August) Siberian precipitation. During a positive phase of the AMV, rainfall significantly increases over the Siberian region in correspondence of the three largest Arctic river watersheds (Ob’, Yenisei and Lena). The identified precipitation increase over Siberia becomes robust and steady within 10 years and is consistent with results from previous observational and model-based studies^5,19,20^. Our results suggest that the detected multidecadal modulation of precipitation over Siberia is induced by the North Atlantic AMV perturbation through a change of the mean atmospheric circulation. Several studies have linked the multidecadal variability in the North Atlantic SST to an anomalous seesaw pattern of geopotential height in the high troposphere over the Northern Hemisphere^20,53,54^. In the present study, the imposed AMV SST perturbation induces a hemispheric-scale wave-train altering upper tropospheric circulation over northern Eurasia. By moisture-flux divergence analysis, we find evidence that Siberian rainfall increase is determined by the combined effect of a westerly flux, carrying moisture from the Atlantic Ocean, along with a southerly moisture flow, from the Pacific basin. Interestingly, previous studies have only detected one of the two moisture transport anomalies. Above-normal advection of moisture from the North Atlantic Ocean has characterized the last decades, transporting it along the northern sector of Eurasia^5,18^, mainly caused by a strong Icelandic Low^19^. On the other hand, S15^20^ claims that the AMV may excite an anomalous anticyclone over Mongolia, leading to a northward moisture transport, in line with our findings and previous studies^53^. Differences in response stress the need of a fully-coupled model framework which accounts ocean feedbacks.

In addition, an analysis of the Siberian rivers’ runoff and potential density over the Arctic Ocean reveals a significant AMV impact on the Arctic freshwater balance via increased river discharge, associated with the local rainfall enhancement, consistently with the observations^44,55^. According to the present model results, the AMV-induced above-normal rainfall contributes to the increased runoff of Ob’, Yenisei and Lena, accelerating the Arctic hydrological cycle.

Low-frequency in-phase relationship is found between river discharge, Eurasian shelves’ stratification and sea-ice summer melt during positive AMV years. The anomalous river runoff weakens the potential density, strengthening local stratification and sub-surface warming. This response is physically consistent with a previous study experiments in which Eurasian river discharge is artificially doubled^48^.

Other local factors, related to the land–atmosphere coupling (not analysed here), may also play a role. For instance, positive winter surface temperature anomalies may influence the snowmelt season, shifting the river discharge peak towards early spring^56^. Another caveat of this work is the assumption of a linear response to AMV, embedded in the adopted DCPP-C experimental protocol. Deviations from the linear hypothesis are not assessed in this study.

The moisture flux analysis presented here focuses on the mean response, thus ignoring the feedback of the transient eddies. While this may represent a shortcoming of this study, it is important to note that the Siberian region is characterized by a much lower eddy activity compared to the oceanic storm-track regions in the North Atlantic and North Pacific sectors^57^. In summer, the contribution of transient eddies may be reduced to a corrective factor which mainly scales the meridional component of the moisture flux^58^, probably increasing the meandering of the total flow.

To conclude, our idealized numerical experiments support a robust connection linking the AMV with climatic variability over northern Eurasia and the Arctic. These results underline that the quality of decadal predictions will substantially benefit from an improved understanding and model representation of the AMV and its related teleconnections.

## Methods

### Data

The AMV spatial pattern and the AMV index are provided by Decadal Climate Prediction Project (DCPP)^22^, also available here: https://esgf-node.llnl.gov/search/input4mips/.

Monthly mean variables have been used in this study, including for the computation of the moisture transports. This implies that the contribution of sub-monthly transient eddies is not accounted for in this study (this point is further examined in “Discussion” section). AMV simulations during the current study are available from the corresponding author on reasonable request.

### CMCC-CM2 Model

To assess the AMV climate impact on global scale, the CMCC–CM2 model is used^59,60^. It is basically grounded on the Community Earth System Model (CESM, https://www.cesm.ucar.edu) while the Nucleus for European Modelling of the Ocean (NEMOv3.6 https://www.nemo-ocean.eu) is used for the ocean component. In this study, the oceanic grid configuration employs the ORCA1 tripolar grid, 1° at the equator, and 50 geopotential vertical levels, ranging from 1 m at the surface to 5,900 m at the ocean bottom. The physical component of the atmospheric model is the Community Atmospheric Model version 5 (CAM5), with a regular, horizontal grid of 0.9° × 1.25° and 30 vertical hybrid levels, 13 of which above 200 hPa to include the lower-stratosphere. The Community Ice CodE is the sea-ice model, sharing the horizontal grid with the ocean in CMCC-CM2. The land component is the Community Land Model version 4.5 (CLM4.5). For this study, the model is used in its "Satellite Phenology" mode, namely the prognostic carbon–nitrogen model is deactivated, and employs the horizontal grid of the atmospheric component. CLM is coupled with the River Transport Model, which routes liquid and ice runoff from the land surface model to the active ocean to simulate a closed hydrologic cycle.

To isolate the internal variability effects and neglect the anthropogenic and external contributions, all external forcings (such as Greenhouse Gases, solar radiation and tropospheric aerosols) are set at the CMIP6 pre-industrial values, constant at 1850’s levels: CO_2_ concentration is 284.32 ppm, CH_4_ concentration is 808.25 ppb and N_2_O concentration is 273.02 ppb.

### Experimental protocol

In order to simulate the AMV impact, the model SST of the North Atlantic Ocean are nudged towards the spatial pattern, strongly following the DCPP experimental protocol. In the ocean component of the CMCC coupled model, NEMO uses flux formulation to globally restore SST, considering a negative feedback term to be added to the surface non-solar heat. To counterbalance the additional flux term introduced by SST restoring during the simulations, sea surface salinity (SSS) is also nudged to the preindustrial-run climatology. The SSS relaxation is used to avoid the progressive alteration of the mean ocean circulation and thermodynamical balance, reducing at least as possible the density anomaly.

We have considered 32 members in order to decrease the internal variability uncertainty. We have performed two suites of idealized experiments where the SST restoring is applied over the North Atlantic (from 10°N to 65°N) while outside this target region, the model is allowed to freely evolve (Fig. S.4):

AMV + experiments: to simulate the positive AMV phase, we restore the SST towards the AMV spatial pattern added to the model climatology.

AMV- experiments: analogous procedure is applied to simulate the negative AMV phase, but the AMV pattern is subtracted to the model climatology.

The integration time of each run is 10 years, allowing to catch the climate response to the AMV input, while longer simulations may lead to the density drift, introduced by the experimental setup^22^.

A more extensive description of the methodology of DCPP-like experiments can be found in the supplementary material and https://www.wcrp-climate.org/experimental-protocol.

## Supplementary information

Supplementary information.
