# Supplementary material for: The impact of the AMV on Eurasian summer hydrological cycle
Source: Sci Rep. 2020 Sep 2;10:14444. doi: 10.1038/s41598-020-71464-2 (PMC7468114; doi:10.1038/s41598-020-71464-2)
Supplement: Supplementary file 1 — Supplementary information. [file 41598_2020_71464_MOESM1_ESM.pdf]

Supplementary material for

The impact of the AMV on Eurasian Summer hydrological cycle

Dario Nicolì, Alessio Bellucci, Dorotea Iovino, Paolo Ruggieri, Silvio Gualdi

To whom correspondence should be addressed. E mail: dario.nicoli@cmcc.it

This file includes:

- **Supplementary figures (S.1-9)**
- **Winter response**
- **Methods: extended description**

Figure S.1

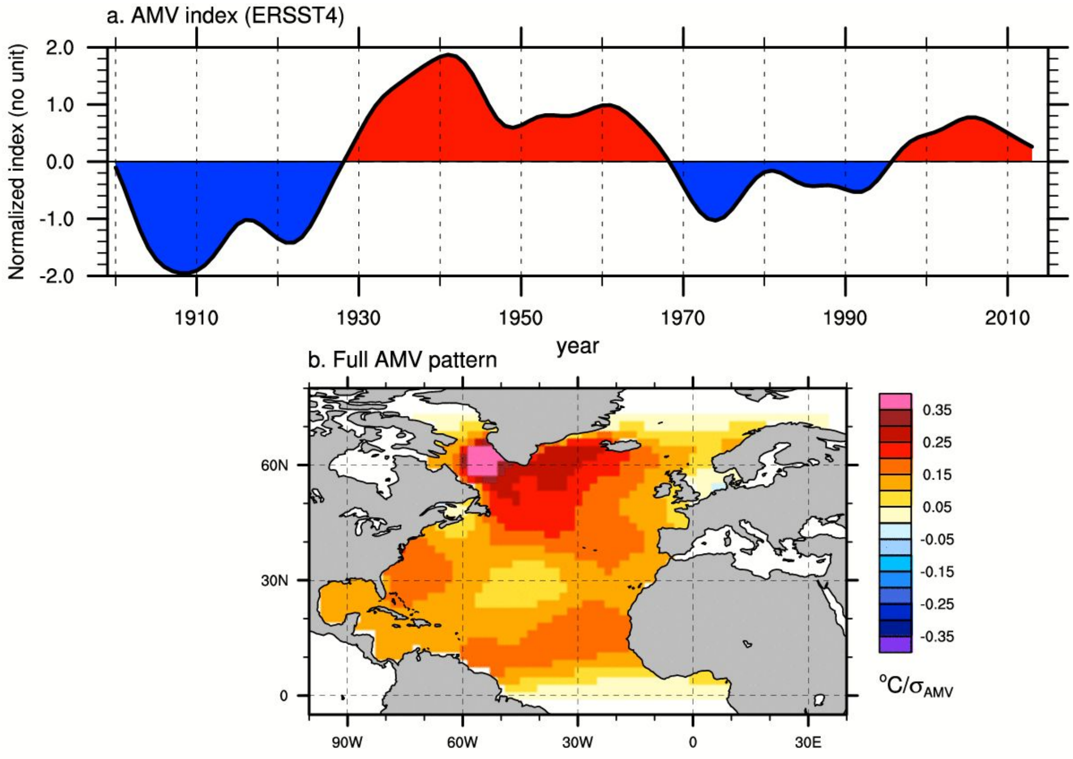

Figure S.1 (top panel) Normalized AMV time series, after filtering as described in DCPP-C Technical Note 1. (https://www.wcrp-climate.org/wgsip/documents/Tech-Note-1.pdf). (bottom panel) AMV SST anomalies obtained after regressing the ERSSTv4 annual residual SST on the AMV index. Contour interval is 0.05°C/σ. The detailed procedure is available in DCPP-C Technical Note 1. [Images from: Technical Note 1, Boer et al. 2016]

Winter response

During Boreal winter, the 2-m temperature response to fixed sea surface temperature (SST) anomalies resembles the AMV pattern over the North Atlantic even if the signal is less significant on the Western side of the basin, along the Gulf Stream. This means that the high heat transport from lower latitudes due to the fast current and the variability of the system is greater than the imposed SST anomalies. Nevertheless, the main features of the AMV are well reproduced over the Atlantic Ocean, including both the maxima over the sub-polar gyre (SPG) and Eastern Tropical Atlantic. Significant warming affects Central America, Tibetan plateau and the Saharan region, with maxima over the Middle East and Indian sub-continent (~0.4°C). The North Pacific Ocean is reminiscent of the IPO-like response, as estimated in DCPP protocol, indeed the structure of the negative signal is not interrupted by anomalies of the opposite sign [Sutton and Hodson 2006, Ting et al. 2011, Barcikowska et al. 2017]. Positive anomalies characterize also the Western Tropical Pacific (WTP), Maritime continent and the Indian Ocean, similar to Boreal summer response, reinforcing the idea that such feature is fed by the positive AMV phase, regardless of the season. The Arctic Ocean response reveals a significant increase of air temperature, consistently with existing literature [e.g. Miles et al. 2014], that results in thinner sea ice pack that is more prone to melt, leading to a smaller sea-ice cover at the end of the melting season. Significant variability emerges over the Mediterranean region with local increased 2-mter temperature, while European regions above 50°N are characterized by more uncertainties.

The sea level pressure (SLP) anomaly field can be considered a proxy of the atmospheric-flow changes and provides a measure of the alteration of the large-scale surface circulation induced by the polarities of the AMV. A broad-scale negative SLP anomaly features the North Atlantic Ocean, as a likely consequence of the Atlantic AMV-related

warming (Figure S.2). Nevertheless, non-significant areas covers the extra-tropical portion of the North Atlantic. This is partly linked to the efficiency of the restoring, especially over the SPG, representing a deep-water formation site. Seawater at the surface of the ocean is intensely cooled by the wind and surface air temperatures. Wind flowing over the water also produces a great deal of evaporation, resulting in an increase in the sea salinity and so in the water-mass density along with the temperature decrease. This process is obviously stronger during the colder season, leading to a deeper mixed layer in winter than in summer since the imposed SST is vertically distributed over a great depth. Thus, it is not so unexpected the model lack of significance due to a less effective SST restoring. The SLP response shows a meridional significant dipole, with a positive lobe over Greenland and a negative one over Central Europe extending over the Atlantic, associated with a statistically-significant precipitation increase (Figure S.2). This pattern bears some resemblance with the negative phase of the North Atlantic Oscillation (NAO), in agreement also with 2-m temperature field, even though only a slight SLP weakening occurs at the Azores High.

In the North Pacific, the Aleutian low is weakened due to the positive SLP anomaly (Figure S.2), allowing the injection of cold air from the Arctic towards North-Western America, via a drop in the mean (cyclonic) advection [Ruprich-Robert et al. 2017]. The Z500 clearly project onto the negative phase of the Pacific-North America (PNA) climate mode, reflecting a two-dipole pattern, with anomalies of similar sign located over the Aleutian Islands and over the South-Eastern U.S. [Barnston and Livezey 1987]. Precipitation changes along with SLP indeed project the entire Pacific Ocean onto the cold phase of the El Niño–Southern Oscillation (ENSO) [Trenberth et al. 1998, Kucharski et al. 2011]. The Eastern Pacific is characterized by an increase of SLP, compensated by the negative anomalies of the Indian and Western-Pacific Ocean, resembling the IPO in its negative

polarity [Zhang and Delworth 2015]. Anomalous cyclonic circulation over the WTP is consistent with the recent findings of Sun et al. [2017] which attribute this below-average SLP to a weakening of subtropical North Pacific westerlies and a strengthening of the Wind-Evaporation-SST mechanism. A positive SLP anomaly is localized over South-Western U.S. with reduced rainfall in both the models (Figure S.2). The AMV-induced temperature gradient shifts northward the Atlantic ITCZ, with enhanced rainfalls over North Brazil and equatorial Africa, while the SPCZ signal is quite noisy and not statistically relevant, with positive anomalies over the Maritime continent.

Figure S.2

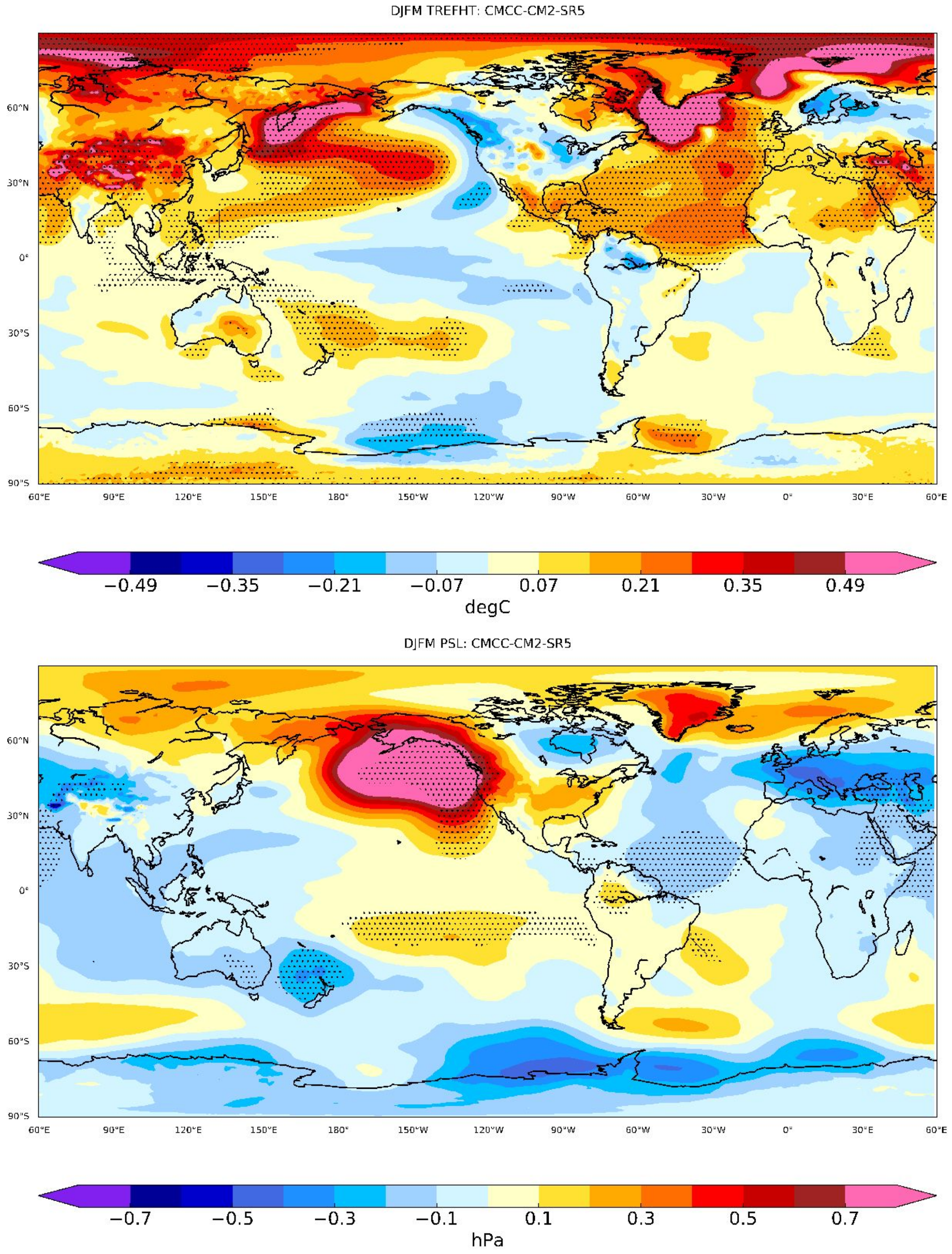
DJFM TREFHT: CMCC-CM2-SR5
60°N
30°N
0°
30°S
60°S
90°S
60°E
90°E
120°E
150°E
180°
150°W
120°W
90°W
60°W
30°W
0°
30°E
60°E
−0.49
−0.35
−0.21
−0.07
0.07
0.21
0.35
0.49
degC
DJFM PSL: CMCC-CM2-SR5
60°N
30°N
0°
30°S
60°S
90°S
60°E
90°E
120°E
150°E
180°
150°W
120°W
90°W
60°W
30°W
0°
30°E
60°E
−0.7
−0.5
−0.3
−0.1
0.1
0.3
0.5
0.7
hPa

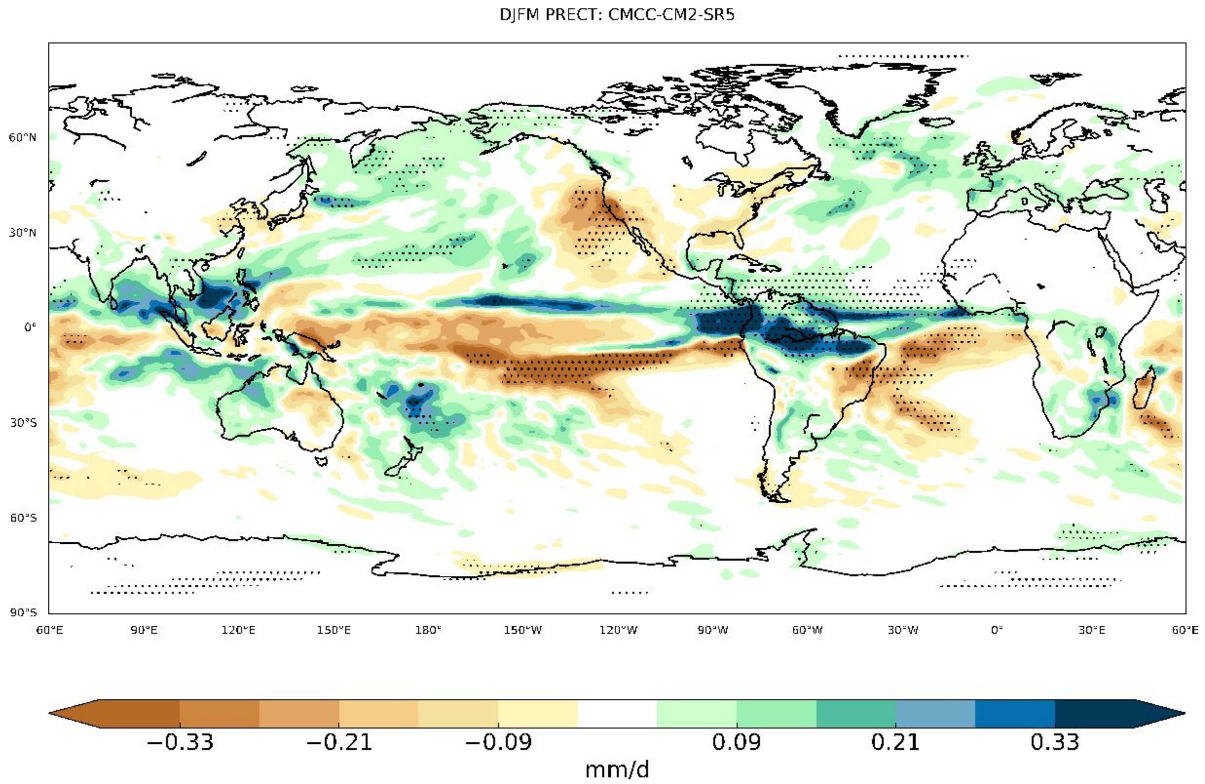

Figure S.2. Boreal winter (DJFM) differences between the last 9-year ensemble mean of the positive and negative phases of the AMV experiments for (a) 2-meter temperature, (b) Sea Level Pressure, (c) Precipitation and (d) . Dotted regions display significant values (Student's t test with 95% confidence level).

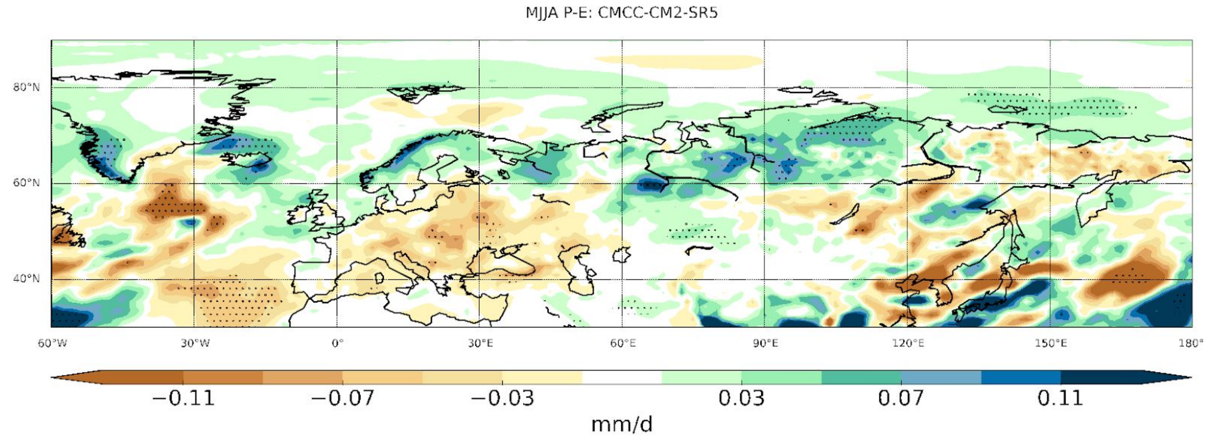

Figure S.3. May-to-August differences between the last 9-year ensemble mean of the positive and negative phases of the AMV experiments for Precipitation-minus-evaporation field. Dotted regions display significant values (Student's t test with 95% confidence level).

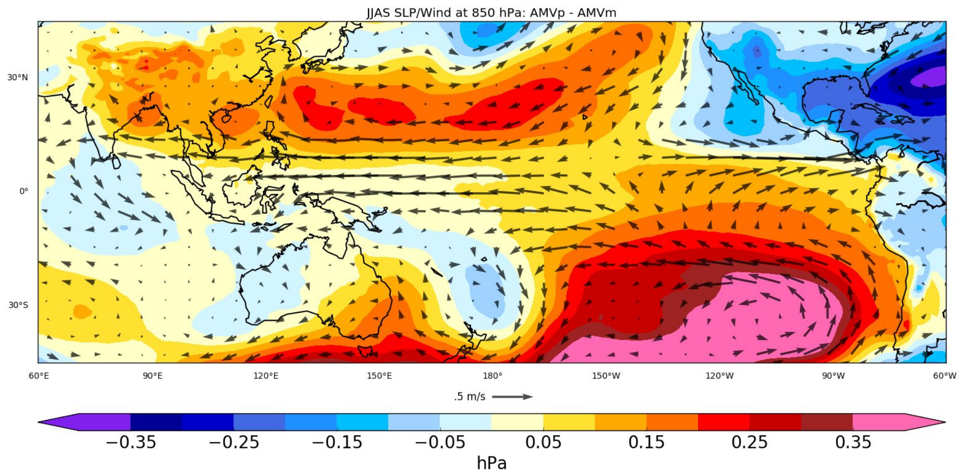

Fig S.5 JJAS difference between the last 9-year ensemble mean of the positive and negative phases of the AMV experiments for sea level pressure (shading, unit is hPa) and lowest-level wind (vectors, unit is m/s)

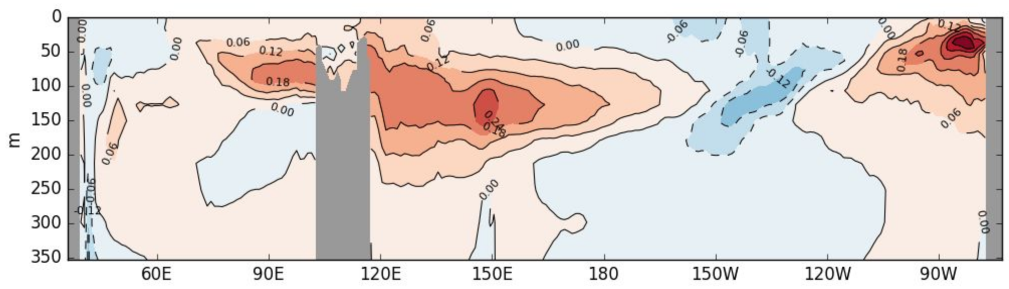

Fig S.6 JJAS differences between the last 9-year ensemble mean of the positive and negative phases of the AMV experiments for ocean temperature (unit is °C) averaged from 5°S and 5°N

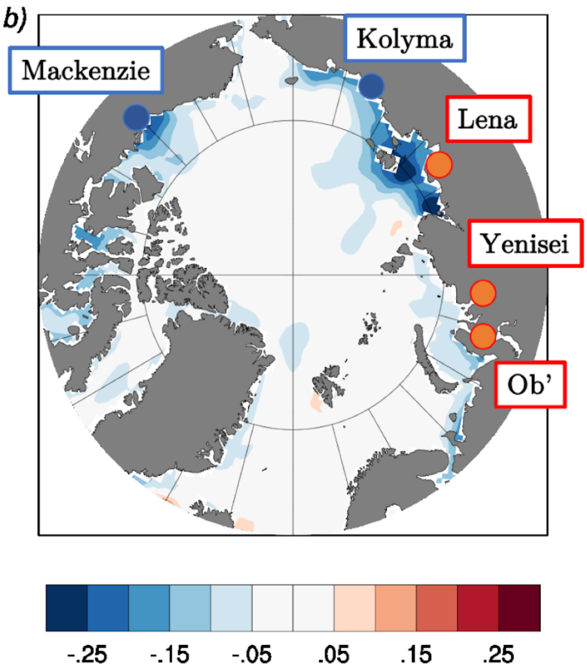

Figure S.7
May-to-August differences between the last 9-year ensemble mean of the positive and negative phases of the AMV experiments for Sea surface salinity [PSU]

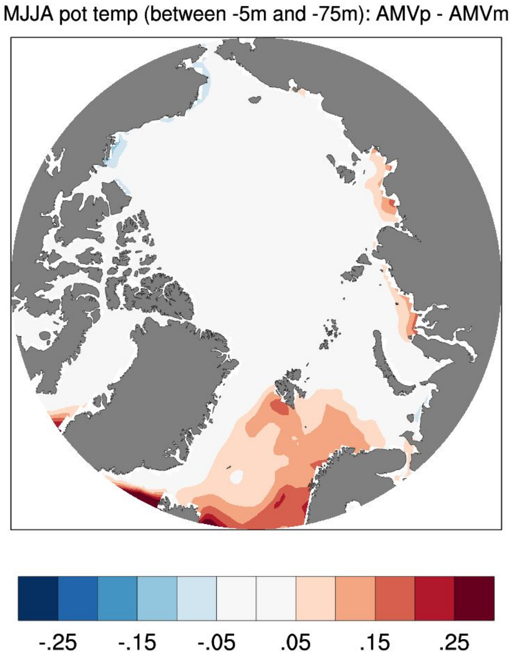

Figure S.8
May-to-August differences between the last 9-year ensemble mean of the positive and negative phases of the AMV experiments for potential temperature [°C], averaged from -5 m and -75 m.

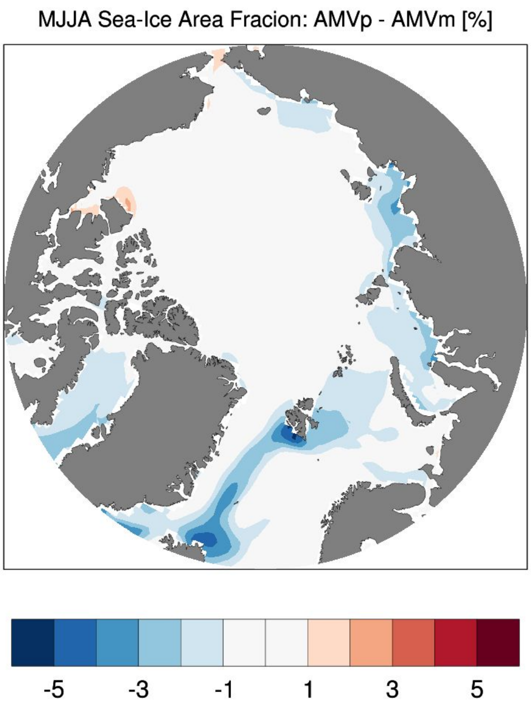

Figure S.9
May-to-August differences between the last 9-year ensemble mean of the positive and negative phases of the AMV experiments for Sea Ice area Fraction [%]

Methods

To assess the AMV climate impact on global scale, the CMCC–CM2 model is used [Cherchi et al. 2018, Fogli and Iovino 2014]. Its core is based on the Community Earth System Model (CESM) except for the ocean component, which is represented by the Nucleus for European Modelling of the Ocean (NEMO). The physical component of the atmospheric model is the Community Atmospheric Model version 5 (CAM5) [Naele et al. 2012]. CAM5 is used in Finite-Volume dynamical-core mode in which the discretization is local and in physical space. The horizontal discretization is based on a conservative “flux-form semi-Lagrangian” scheme [Lin and Rood 1996, Lin and Rood 1997], while the vertical discretization is defined as "quasi-Lagrangian", i.e. a Lagrangian scheme with a conservative re-mapping. All prognostic variables are updated by the dynamics and subsequently by the physics. CAM5 is

characterized by a regular, horizontal grid of 0.9° x 1.25°. The vertical resolution encompasses 30 vertical hybrid levels, 17 of which below 200 hPa to include the lower-stratosphere. The ocean component is NEMOv3.6 [Madec et al. 2016] which is a three dimensional, free-surface, hydrostatic, primitive-equation global ocean general circulation model. In this study, the grid configuration employs ORCA tripolar grid – a global curvilinear ocean grid which has two poles in the northern hemisphere located over land [Madec and Imbard 1996]. The horizontal mesh has a 1° (~100 km) horizontal resolution with a meridional refinement up to 1/3° in the tropical region. The vertical grid has 50 geopotential levels, ranging from 1 m at the surface to 5900 m at the ocean bottom. The Community Ice CodE is the sea-ice model [Hunke and Lipscomb, 2008], sharing the horizontal grid with the ocean in CMCC-CM2. The land component is the Community Land Model version 4.5 (CLM4.5) [Oleson et al. 2013], which is able to simulate the physical, chemical and biological processes involving land surface. For this study, the model is used in its "Satellite Phenology" mode, namely the prognostic carbon–nitrogen model is deactivated, and employs the horizontal grid of the atmospheric component. CLM is coupled with the River Transport Model, which routes liquid and ice runoff from the land surface model to the active ocean to simulate a closed hydrologic cycle [Branstetter and Famiglietti 1999].

To isolate the internal variability effects and neglect the anthropogenic and external contributions, all external forcings (such as Greenhouse Gases, solar radiation and tropospheric aerosols) are set at the CMIP6 pre-industrial values, constant at 1850's levels: CO2 concentration is 284.32 ppm, CH4 concentration is 808.25 ppb and N2O concentration is 273.02 ppb.

In order to simulate the AMV impact, the model SST of the North Atlantic Ocean are nudged towards the spatial pattern. In the ocean component of the CMCC coupled model, NEMO

uses flux formulation to globally restore SST, considering a negative feedback term to be added to the surface non-solar heat flux Q_{ns}^{0} :

$$Q_{ns} = Q_{ns}^{0} + \frac{dQ}{dT}(SST_{MODEL} - SST_{TARGET})$$

where:

- Q_{ns} is the resulting surface non-solar heat flux.
- $\frac{dQ}{dT}(SST_{MODEL} - SST_{TARGET})$ is defined as the heat flux restoring term (hereafter hfcorr) which provides a measure of the restoring efficiency.
- $\frac{dQ}{dT}$ is a negative feedback coefficient (©$_{T}$) which indicates the restoring strength. According to the DCPP protocol, this term is fixed at -40 W m^{-2} K^{-1} equal to a relaxation time scale (|$_{R}$) of 60 days for a 50m Mixed-Layer Depth (MLD).
- SST_{MODEL} is the model SST.
- SST_{TARGET} is the target SST which is, in this study, the positive (or negative) 1 standard deviation of the AMV anomalies superimposed to the model climatology.

Temperature restoring introduces density anomalies over the SPG: for instance, during a simulated positive phase, warm anomalies tends to stabilize the ocean column reducing the deep ocean convection and generating a weakening of the subpolar gyre (SPG) density. These negative density anomalies may induce a decline in the AMOC and SPG strength, also affecting the poleward ocean heat transport. In the opposite case, imposing cold anomalies over the SPG can lead to a surplus of poleward ocean heat transport [Boer et al. 2016, Ruprich-Robert et al. 2017]. This drift is strongly model dependent [Tech. Note II, Boer et al 2016].

To reduce the non-linear uncertainty related to the internal variability of the climate system, an ensemble approach is used. The DCPP protocol recommends to generate the ensemble

using at least 25 "macro-perturbations" [Hawkins et al. 2016], i.e. considering 25 different ocean states as initial condition of each realization, in addition to only different atmospheric states ("micro-perturbation"). This ensures a better spread among the ensemble members. In this study, 32 members are considered taken from a multicentury pre-industrial run. To counterbalance the additional flux term introduced by SST restoring during the simulations, sea surface salinity (SSS) is also restored to the preindustrial-run climatology. The SSS relaxation is used to avoid the progressive alteration of the mean ocean circulation and thermodynamical balance, reducing at least as possible the density anomaly. SSS restoring can be considered as a flux correction on freshwater fluxes which has no physical meaning. As for the SST, a flux formulation is used to restore SSS adding a feedback term in the freshwater budget to the freshwater flux EMP:

$$EMP = EMP^0 + \gamma_S \frac{(SSS_{MODEL} - SSS_{TARGET})}{SSS_{MODEL}}$$

where:

- EMP is the resulting freshwater flux (evaporation minus precipitation).
- SSS_{TARGET} is the climatological SSS for both the AMV phases.
- SSS_{MODEL} is the model SSS.
- γ_S is the negative SSS restoring term which indicates the magnitude of the damping in the salinity. Unit is mm/d.

Given the model-dependence nature of γ_S, a tuning of salinity damping term has been necessary to assess the best value which contributes to perturb the least possible the climate-system equilibrium. According to hfcorr diagnostics, setting γ_S equal to –432.00 mm/d ($T_R \cong 4$ months for a 50-m MLD) determines a better restoring in terms of SST convergence towards the observed AMV phase, without excessively altering the ocean circulation and preserving the characteristics of the water masses.

The SST/SSS restoring is applied over the North Atlantic (from 10°N to 65°N) based on a mask provided by DCPP protocol on a 2-degree horizontal grid. Outside this target region, the model is allowed to freely evolve. Following the protocol [Boer et al. 2016], the original AMV mask is extrapolated over land to minimize the interpolation errors and, using a bilinear method, is regridded onto the ORCA1 tripolar grid of NEMO ocean model (Figure S.4). An 8-degree wide buffer zone is opportunely designed at the edge of the nudging area to minimize shocks and to avoid instabilities in the no-restoring region.

To reduce the non-linear uncertainty related to the internal variability of the climate system, an ensemble approach is used. The DCPP protocol recommends to generate the ensemble using at least 25 "macro-perturbations" [Hawkins et al. 2016], i.e. considering 25 different ocean states as initial condition of each realization, in addition to only different atmospheric states ("micro-perturbation"). This ensures a better spread among the ensemble members. In this study, 32 members are considered taken from a multicentury pre-industrial run.

Two sets of idealized experiments have been performed where the perturbation via restoring is applied:

- AMV+ experiments: North Atlantic (10°N – 65°N) SSTs are restored to positive time-independent AMV anomaly (i.e. +1 standard deviation of the AMV index) superimposed on 12-month model climatology. No restoring is performed where the sea ice fraction is greater than 15%, which is the Sea-Ice Extent definition.
- AMV- experiments: they are analogous to AMV+ experiments, but it is considered the negative AMV anomaly (i.e. -1 standard deviation of the AMV index).

Each simulation is integrated over a 10-year period, allowing to catch the climate response to the AMV input, while longer simulations may lead to the aforementioned drift, introduced by the experimental setup [Ruprich-Robert et al. 2017].

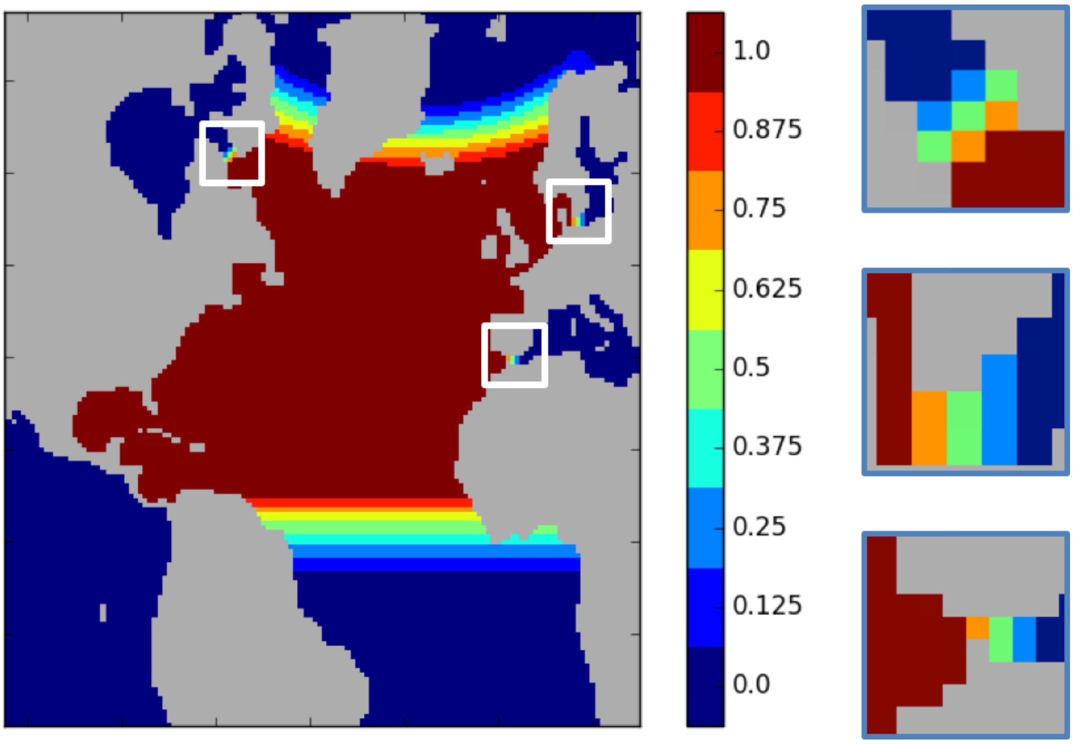

Figure S.4: (left) Mask used to restore the SST in the North Atlantic on ORCA1 tripolar grid and (white squares in the left panel; magnified on the right panels) the critical buffer areas: (top) the Hudson strait in the Labrador Sea, (mid) the Danish strait in the Baltic Sea and (bottom) the Gibraltar strait in the Mediterranean Sea. The buffer zone increases with a step of 0.125 per latitude degree from zero (no restoring) to one (full restoring).
